# The association between retinal vessel abnormalities and H-type hypertension

**DOI:** 10.1186/s12883-020-02029-z

**Published:** 2021-01-06

**Authors:** Kuankuan Huang, Zhixiang Zhang, Shan Huang, Yanwen Jia, Min Zhang, Wenwei Yun

**Affiliations:** 1 Department of Neurology, Changzhou No.2 People’s Hospital Affiliated to Nanjing Medical University, No.29, Xinglong Lane, Tianning District, Changzhou, 213004 Jiangsu China; 2 Ophthalmology Department of Changzhou No.2 People’s Hospital Affiliated to Nanjing Medical University, No.29, Xinglong Lane, Tianning District, 213004 Changzhou, Jiangsu China

**Keywords:** Retinal vessel abnormality, Homocysteine, Hypertension

## Abstract

**Background:**

This study aimed to investigate the relationship between H-type hypertension and retinal vessel abnormalities.

**Methods:**

Hypertensive patients were retrospectively enrolled in this study. According to plasma homocysteine (HCY), patients were divided into isolated hypertension and H-type hypertension groups. The diameter of retinal vessels and retinopathy were evaluated by retinal fundus photography. The differences of retinal vessel abnormalities between H-type hypertension and isolated hypertension were investigated by univariate and multivariate regression.

**Results:**

A total of 191 hypertensive patients were included, of which 86 were with isolated hypertension and 105 with H-type hypertension. The H-type hypertension group had a higher ratio of retinopathy(*P* = 0.004) and higher degree of retinal arteriosclerosis (*P* = 0.005) than the isolated hypertension group. CRAE (107.47 ± 13.99µ m vs. 113.49 ± 11.72µ m, *P* = 0.002) and AVR (0.55 ± 0.06 vs. 0.58 ± 0.06, *P* = 0.001) were smaller in H-type hypertension group than those in isolated hypertension group. Multivariate analysis showed that after adjusting for age, sex, course of hypertension and diabetes, H-type hypertension was an independent risk factor of retinopathy (OR, 2.259; 95%CI, 1.165—4.378; *P* = 0.016), CRAE (β=-5.669; 95%CI, -9.452—-1.886; *P* = 0.004), and AVR (β=-0.023; 95%CI, -0.039—-0.007; *P* = 0.005).

**Conclusions:**

H-type hypertension is closely related to more retinal vessel abnormalities than isolated hypertension. Controlling H-type hypertension may reduce the risk of small vascular damage.

## Background

Homocysteine (HCY) is an important intermediate product in methionine metabolism and elevated HCY levels can enhance the risk of cerebral small vessel diseases [1–3]. Compared to isolated hypertension, hypertension with elevated plasma HCY, which is defined as H-type hypertension, may further aggravate the cerebral small vascular damage. Retinal vessels, the diameter of which is approximately 100 µm to 200 µm, are quite sensitive to hypertension and closely related to cerebral small vessel diseases [4]. There are many methods and techniques to evaluate small vascular damage, such as retinal fundus photography, video-capillaroscopy, near infrared spectroscopy and side-stream dark field and so on. Retinal fundus photography is widely used in clinic which can directly assess the retinal vessels.

However, research on the relationship between H-type hypertension and retinal vessels remains scarce. In this study, we evaluated the retinal vessel abnormalities using retinal fundus photography as a non-invasive examination. Specifically, diameters of the retinal vessels were measured, and the degree of retinal arteriosclerosis and other retinal lesions were visually rated according to the retinal fundus photographs. The relationship of H-type hypertension and retinal vessel abnormalities was investigated.

## Methods

### Patients

The study was approved by the Institutional Ethics Committees of the Changzhou No.2 People’s Hospital Affiliated to Nanjing Medical University (NO. 2018-KY032-01). Informed consent was obtained from all participants. All the patients were recruited from February 2019 to August 2019 through the Department of Neurology at Changzhou No.2 People’s Hospital. Patients who diagnosed essential hypertension and aged 18 years or older were enrolled. Exclusion criteria were as follows: (1) patients who were not able to provide informed consent; (2) unavailable or unreliable retinal fundus photographs (e.g. due to poor imaging quality); (3) patients with serious complications such as organ failure, serious infections and so forth; (4) patients who had undergone eye surgery (such as cataract extraction, laser surgery, glaucoma surgery, pseudo eyeball replacement, etc.) in the past six months.

### H-type hypertension diagnosis

Patients enrolled into the H-type hypertension group were diagnosed with the combination of hyperhomocysteinemia (HHCY) and hypertension. The criteria of hypertension are based on their past medical history or blood pressure ≥ 140 mmHg SBP and/or ≥ 90 mmHg DBP without medicine. HHCY is defined as plasma HCY more than or equal to 10 µmol per liter. A noninvasive ambulatory blood pressure monitor (model 6100) was used to measure blood pressure. The purpose and precautions of the examination were explained to the patients and their families, and their informed consents were obtained. The effective measurement was more than 80 percent of the measurement times. The monitoring starts and ends at 06:00–06:00 of the next day, 15 min/time in the daytime (06:00–22:00), and 30 min/time at night (22:00–06:00 of the next day). The 24-hour mean systolic and diastolic pressures were calculated by a computer. To measure plasma HCY, 4 ml peripheral venous blood was collected in the morning and placed in the heparin anticoagulant tube (Shanghai Hengyuan biological technology co., LTD.). The blood was centrifuged at 4000r/min (ebende ag., Germany) for 5 min to separate the plasma. The plasma HCY was detected by Siemens ADVIA2400 automatic biochemical analyzer. Other related clinical characteristics and demographics were collected in the hospital. More information including demographic, clinical and laboratory factors were collected.

### Retinal fundus photograph assessment

**Fig. 1:**
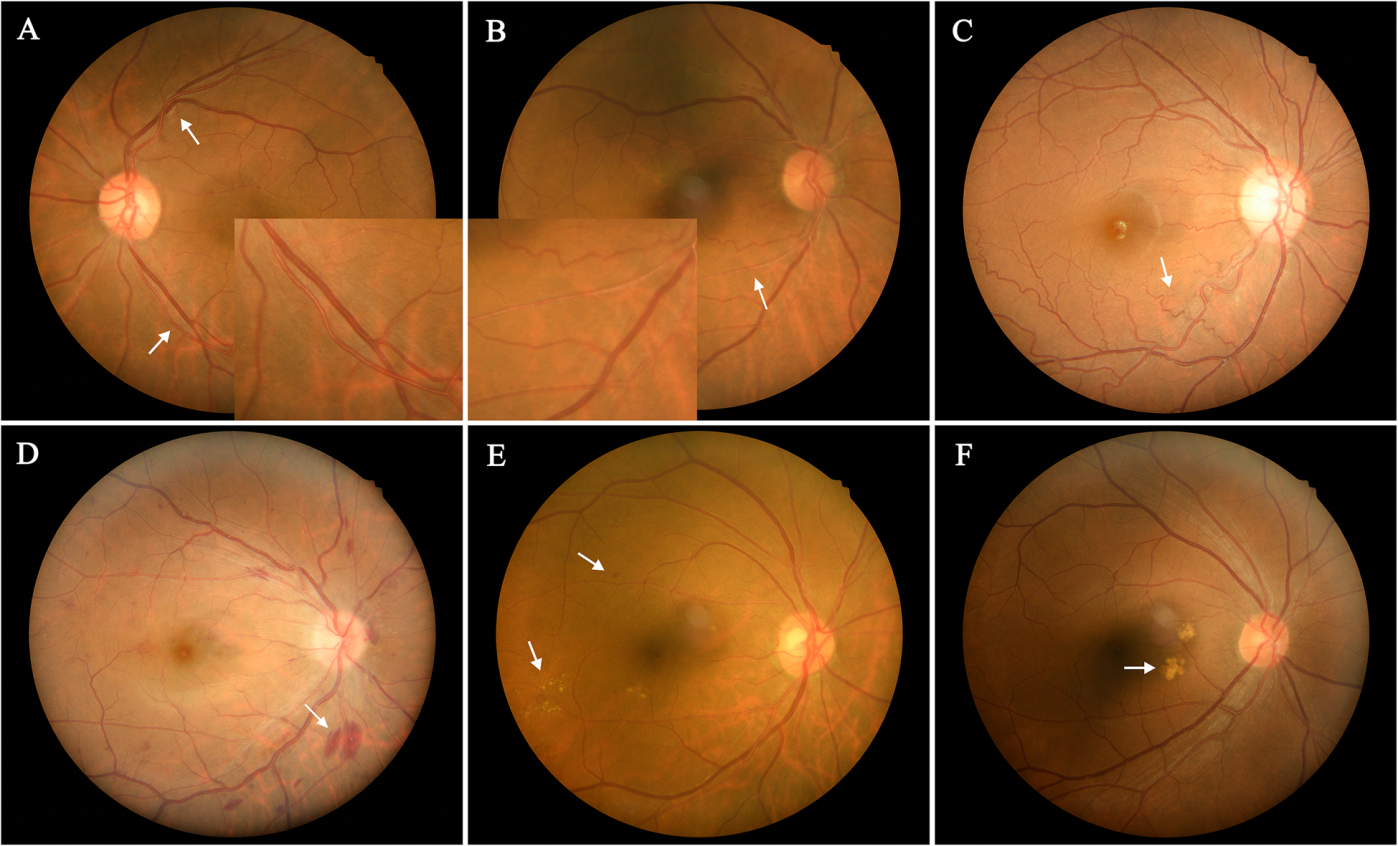
Retinal fundus photographs of patients. **a**, Arteriovenous nicking (upper arrow), normal retinal artery (magnified at the lower arrow); **b**, Retinal arteriosclerosis (magnified at the arrow), The retinal artery becomes thinner, the reflection is enhanced, and the blood column becomes lighter in color; **c**, Retinal artery is widely wavy (arrow point). **d**, Hemorrhage of the retina (arrow point); **e**, Microangioma (upper arrow), hard exudation (lower arrow); **f**, Soft exudate (arrow point)

Several classic abnormalities of hypertensive ophthalmopathy were used to evaluate retinal lesions through retinal photographs. Retinopathy was defined as present if any of the following retinal vascular abnormalities were detected through retinal fundus photographs: retinal hemangioma, hemorrhages, hard and soft exudates. Arteriovenous nicking (AVN), the curvature of vessels and arteriosclerosis grades had also been rated. Retinal arteriosclerosis grades were assessed according to Scheie grading method [5]. And diameters of retinal vessels were also measured (Fig. 1).

All patients received 45-degree color photos of the retinal fundus with Kowa nonmyd Wx nonmydriatic fundus camera. Make sure that the optic disc was in the center, and retinal fundus photography was performed. The pictures were saved in TIFF format (pixels: 2992 × 2000). Image J software was used to measure the diameter of six retinal vessels. First, we magnified the fundus photograph obtained by the same multiple, and then circled the range of 1∕2–1 papilla disc (PD) from the edge of the optic disc. Third, we selected the edge of the blood vessel wall with the mouse, measuring the diameter of the blood vessel vertically. The software automatically displayed the measurement results [5–7]. Repeat the above operation to get the average value of each vessel diameter to reduce the artificial errors. Thus, we got the diameter of six retinal arteries (Wa1–Wa6) and retinal veins (Wv1–Wv6) together with the vertical diameter (PD1) and horizontal diameter (PD2) of the optic disc at the same time. PD is equal to half of the sum of PD1 and PD2. The whole measurement process was completed by a graduate student. When measuring, we try to avoid the vascular bifurcation. After that, the measured data unit was a pixel. The average diameter of the optic disc is close to 1850 micrometers (µm). We had to convert its’ unit from pixel into µm.

$$ \mathrm{W}\left(\mathrm{actual}\right)=1850/\mathrm{PD}\times \mathrm{W}\left(\mathrm{measurement}\right) $$

Thus, the actual diameters of six retinal arteries and retinal veins were obtained respectively. Then, we used an iterative operation formula named Parr-Hubbard formula to get central retinal vein equivalent (CRVE), central retinal artery equivalent(CRAE) and arteriole-to-venule ratio(AVR) [6]. The formula is as follows:

$$ \mathrm{CRVE}=0.95\times {\left({{\mathrm{W}}_{\mathrm{min}}}^2+{{\mathrm{W}}_{\mathrm{max}}}^2\right)}^{1/2} $$

$$ \mathrm{CRAE}=0.88\times {\left({{\mathrm{W}}_{\mathrm{min}}}^2+{{\mathrm{W}}_{\mathrm{max}}}^2\right)}^{1/2} $$

$$ \mathrm{AVR}=\mathrm{CRAE}/\mathrm{CRVE} $$

### Statistical analysis

Statistical analysis was performed by SPSS Statistics Version 25. Continuous variables with normal distribution were expressed as mean ± SD. Those with non-normal distribution were expressed as median (interquartile range). Categorical variables were expressed as the number of cases (proportion). The differences of baseline characteristics between H-type hypertension and isolated hypertension group were described. All patients were divided into two groups according to the presence of retinopathy. Differences were determined by χ^2^, T test or Mann Whitney U where appropriate. Univariable and multivariable logistic regression were used to estimate the association between H-type hypertension and retinopathy. We also used univariable and multivariable linear regression to explore the relation between H-type hypertension and CRAE, H-type hypertension and AVR.

## Results

### Baseline characteristics of isolated and H-type hypertension

A total of 191 hypertensive patients were enrolled in this study. The average of total plasma HCY for patients is 13.83 ± 9.99µ mol/L. Table 1 outlines relevant characteristics of hypertension and H-type hypertension groups. H-type hypertension group had a higher proportion of retinopathy (*P* = 0.004) and higher grades of retinal arteriosclerosis (*P* = 0.005). There was a significant difference in arterial diameter between the two groups. CRAE (*P* = 0.002) and AVR (*P* = 0.001) were smaller in H-type hypertension group (Table 1).

**Table 1:** Baseline characteristics of patients of two groups (*N* = 191)

Variable	Isolated hypertension (86)	H-type hypertension (105)	*P* value
Characteristics			
Age, y, mean ± SD	64.62 ± 10.97	66.29 ± 12.35	0.328
Sex, male, n (%)	63(73.3%)	76(72.4%)	0.892
Smoking currently, n (%)	41(47.7%)	53(50.5%)	0.700
Alcohol use, n (%)	17(19.8%)	29(27.6%)	0.207
Atrial fibrillation, n (%)	8(9.3%)	4(3.8%)	0.120
Coronary heart disease, n (%)	8(9.3%)	12(11.4%)	0.633
Diabetes, n (%)	23(26.7%)	39(37.1%)	0.127
Hypertension duration, median (IQR)	10.00(5.0,13.00)	12.00(6.00,15.00)	0.145
Laboratory examination, Mean ± SD			
Total cholesterol, mmol/L	3.92 ± 1.03	4.01 ± 0.78	0.489
Triglyceride, mmol/L	2.07 ± 3.73	1.75 ± 0.87	0.400
LDL, mmol/L	2.09 ± 0.75	2.21 ± 0.64	0.215
HDL, mmol/L	1.05 ± 0.31	1.05 ± 0.25	1.000
Serum creatinine, mmol/L	69.90 ± 19.95	70.75 ± 17.41	0.755
Homocysteine, µmol/L, median (IQR)	8.50(7.33, 9.33)	14.25(12.23,18.37)	0.000
Retinal vessel abnormalities			
Retinopathy, n (%)	19 (22.1%)	44 (41.9%)	0.004
Hemangioma, n (%)	14(16.3%)	26 (24.8%)	0.152
Hemorrhages, n (%)^a^	1(1.2%)	5 (4.8%)	0.316
Hard exudates, n (%)	9(10.5%)	21(20.0%)	0.072
Soft exudates, n (%)	6(7.0%)	11(10.5%)	0.398
Arteriovenous nicking, n (%)	32(37.2%)	53(50.5%)	0.066
Vascular curvature, median (IQR)	1.00(1.00,1.00)	1.00(1.00,1.50)	0.511
Arteriosclerosis grades, median (IQR)	1.00(0.00,1.00)	1.00(0.00,2.00)	0.005
CRVE,µm	195.98 ± 21.19	194.07 ± 19.10	0.515
CRAE,µm	113.49 ± 11.72	107.47 ± 13.99	0.002
AVR	0.58 ± 0.06	0.55 ± 0.06	0.001

*Abbreviations*: *SD* standard deviation, *IQR* interquartile range, *LDL* low-density lipoprotein, *HDL* high-density lipoprotein, *CRVE* central retinal vein equivalent, *CRAE*, central retinal artery equivalent, *AVR* arteriole-to-venule ratio

^a^Continuity correction were used

### Baseline characteristics of factors of retinopathy

Table 2 shows the results of related risk factors of retinopathy. The proportion of patients with H-type hypertension in retinopathy group [44(69.8%)] was higher than that in the group without retinopathy [61(47.7%)]. The difference was significant between the two groups (*P* = 0.004). Other risk factors, such as history of diabetes(*P* = 0.013), courses of hypertension (*P* = 0.032) and age (*P* = 0.024) were also the decisive factors leading to the incidence of retinopathy.

**Table 2:** Baseline characteristics of retinopathy

Variable	Without Retinopathy (128)	With Retinopathy (63)	*P* value
Age, y, mean ± SD	64.20 ± 12.09	68.25 ± 10.47	0.024
H-type hypertension group, n (%)	61(47.7%)	44(69.8%)	0.004
male, n (%)	98(76.6%)	41 (65.1%)	0.094
Smoking currently, n (%)	67(52.3%)	27 (42.9%)	0.218
Alcohol use, n (%)	32(25.0%)	14 (22.2%)	0.673
Atrial fibrillation, n (%)	7(5.5%)	5 (7.9%)	0.509
Coronary heart disease, n (%)	11 (8.6%)	9 (14.3%)	0.227
Diabetes, n (%)	34(26.6%)	28 (44.4%)	0.013
Hypertension duration, median (IQR)	10.00(5.00,13.00)	12.00(8.00,16.00)	0.032
Hypertension grades			0.637
Grade 1 n (%)	8(6.3%)	6(9.5%)	
Grade 2 n (%)	48(37.5%)	25(39.7%)	
Grade 3 n (%)	72(56.3%)	32(50.8%)	
SBP, mmHg, mean ± SD	145.84 ± 22.48	147.35 ± 17.13	0.603
DBP. mmHg, mean ± SD	85.47 ± 12.88	83.54 ± 12.15	0.323
Total cholesterol, mmol/L	3.95 ± 0.92	4.00 ± 0.87	0.732
Total cholesterol, mmol/L	2.02 ± 3.11	1.65 ± 0.76	0.352
LDL, mmol/L	2.14 ± 0.70	2.19 ± 0.68	0.690
HDL, mmol/L	1.05 ± 0.29	1.05 ± 0.24	0.932
Serum creatinine, mmol/L	69.54 ± 18.48	72.06 ± 18.73	0.379
HCY, µmol/L, median (IQR)	9.96(8.42,13.80)	13.42(9.10, 20.23)	0.001
HCY levels, n (%)			0.001
Normal (5.04 to 9.99, µmol/L)	64(50%)	21(33.3%)	
Mild (10.00 to 14.99, µmol/L)	44(24.4%)	17(27.0%)	
Severe (15.00 to 65.00, µmol/L)	20(15.6%)	25(39.7%)	

*Abbreviations*: *SD* standard deviation, *IQR* interquartile range, *SBP* Systolic blood pressure, *DBP* diastolic blood pressure, *HCY* homocysteine, *LDL* low-density lipoprotein, *HDL* high-density lipoprotein

Retinopathy was defined as present if any of the following retinal vascular abnormalities were detected through retinal fundus photographs: retinal hemangioma, hemorrhages, hard exudates and soft exudates

Hypertension grades: Grade 1, SBP:140 to 159 mmHg and/or DBP: 90 to 99 mmHg; Grade 2, SBP:160 to 179 mmHg and/or DBP: 100 to 109 mmHg; Grade 3, SBP ≥ 180 mmHg and/or DBP ≥ 110 mmHg. The standards of the grading are based on the highest blood pressure without medicine since the diagnosis of hypertension

### Logistic regression between H-type hypertension and retinopathy

Univariable logistic analysis showed that H-type hypertension was related to retinopathy (OR, 2.544; 95%CI, 1.341—4.825; *P* = 0.004, Table 3). After adjusting for age, sex, history of diabetes and course of hypertension, presence of H-type hypertension remained significantly associated with retinopathy (OR, 2.259; 95%CI, 1.165—4.378; *P* = 0.016).

**Table 3:** Univariable and multivariable logistic regression between H-type hypertension and retinopathy

Variable	OR (95%CI)	*P* value
Crude	2.544(1.341—4.825)	0.004
Model 1	2.477(1.293—4.745)	0.006
Model 2	2.259(1.165—4.378)	0.016

Crude: Univariable logistic regression analysis of H-type hypertension and retinopathy

Model 1: Adjusting for age, sex

Model 2: Adjusting for age, sex, history of diabetes, courses of hypertension

### Linear regression between H-type hypertension and diameters of retinal vessels

H-type hypertension was associated with CRAE by univariate linear analysis (β, -6.023;95%CI, -9.759—-2.288; *P* = 0.002, Table 4). After adjusting for age, sex, history of diabetes and courses of hypertension step by step, H-type hypertension was independent risk factor of CRAE (β, -5.669; 95%CI, -9.452—-1.886; *P* = 0.004). An analogous correlation was observed between H-type hypertension and AVR. H-type hypertension was associated with a decrease in AVR (β, -0.023; 95%CI, -0.039—-0.007; *P* = 0.005).

**Table 4:** Univariable and multivariable linear regression between H-type hypertension and retinal vessel calibers

Variable	CRAE			AVR		
	β	95%CI	*P* value	β	95%CI	*P* value
Crude	-6.023	-9.759—-2.288	0.002	-0.027	-0.044—-0.011	0.001
Model 1	-5.934	-9.690—-2.178	0.002	-0.027	-0.043—-0.010	0.002
Model 2	-5.669	-9.452—-1.886	0.004	-0.023	-0.039—-0.007	0.005

Crude: Univariable linear regression analysis of H-type hypertension and retinal vessel calibers

Model 1: Adjusting for age, sex

Model 2: Adjusting for age, sex, history of diabetes, courses of hypertension

*Abbreviations*: *CRAE* central retinal artery equivalent, *AVR* arteriole-to-venule ratio

## Discussion

This study found that H-type hypertension tended to have more severe retinal vessel abnormalities than isolated hypertension. Patients with H-type hypertension had smaller retinal arteries and more severe retinal arteriosclerosis than isolated hypertension.

H-type hypertension was first proposed by a Chinese research team in 2008 [8]. It refers to patients with hypertension and HHCY at the same time, and HHCY is defined as HCY over 10 µmol/L [9–12]. H-type hypertension accounts for a high proportion of hypertension, approximately 75% [13]. Previous studies found that HCY is another major risk factor for cerebrovascular disease in addition to hypertension. A meta-analysis also found that hypertension was closely associated with HCY, and there is a direct interaction between hypertension and HCY [14]. In our study, the median value of HCY in H-type hypertension group was 14.25 µmol/L (interquartile range, 12.23 to 18.37 µmol/L). There are several reasons for elevated HCY. Metabolic disorders are the most important reason among all. HCY has three main metabolic pathways. One of the metabolic pathways is that HCY is catalyzed by vitamin B6 dependent cystathionine β synthetase. In this process, HCY is converted to cysteine and eventually metabolizes in the body to produce H_2_S. Accumulating evidence indicated that H_2_S is a physiological vasorelaxant and reduced production of H_2_S in the vascular tissue leads to hypertension [15–17]. Besides, HHCY may cause direct toxicity and vascular endothelial injury, which may induce hypertension or aggravating the damage of hypertension to vessels [10, 18, 19]. Collectively, HCY also activates certain metalloproteinases which can cause degradation of collagen and elastin leading to vascular hypertrophy [20, 21]. Moreover, the accumulation of HCY leads to increased cellular oxidative stress in which mitochondrial thioredoxin and peroxiredoxin are decreased and NADH oxidase activity is increased [15]. In a word, HHCY can induce arteriosclerosis through several mechanisms as mainly includes endothelial cell injury, oxidative stress and vascular remodeling.

Several previous studies have investigated the relationship between HCY and vascular disease. Sottilotta et al. found that elevated plasma homocysteine could be an independent risk factor of retinal vascular occlusive disease which is closely related to coronary, cerebral, and peripheral atherosclerotic vascular disease [22]. A study also the evaluated the availability of HCY as a biomarker for diabetic retinopathy [23]. In our study, patients in H-type hypertension had more serious retinal vessel abnormalities lesions compared with isolated hypertension. Further analysis demonstrated that although the related risk factors were adjusted, H-type hypertension remained a risk factor of retinopathy. The mechanism of damage of HCY to small arteries is not completely clear, but the endothelium damage of small vessels by HCY may be an important factor. When HCY and hypertension coexist at the same time, the damage of small vascular is often more serious. The pathological changes of the retinal vessels are regarded as the window of systemic small vascular problems. The parameters of retinal vessel abnormalities include the grade of retinal arteriosclerosis, diameter of retinal artery, AVN and so on. We found that patients with H-type hypertension had a higher grade of retinal arteriosclerosis, and smaller diameter of retinal artery compared with isolated hypertensive patients. The changes of retinal arteriosclerosis include thinning and straightening of the retinal artery, which is like copper wire or silver wire. Retinopathy described above was closely related to the damage of the retinal vessels. In severe cases, retinal vessel spasm, stenosis, obstruction, and hemorrhages can occur. In our study, retinal arteriosclerosis grades, CRAE and AVR seemed to be more sensitive compared with other lesions. AVN barely failed to attain statistical significance. And differently from veins, retinal arteries, instead of veins, showed differences between the two groups. Based on these findings, it is indicative that a higher level of plasma HCY may aggravate retinal vessel abnormalities. Studies also found that H-type hypertension have more severe damage on cerebral small vessels compared with isolated hypertension [24, 25]. Early diagnosis and intervention of H-type hypertension are significantly important in clinic.

Many researchers are interested in HCY [13, 26, 27]. Retinal vessels are the unique small vessels that can be directly observed throughout the body. We measured the diameters to evaluate the changes of retinal vessels in hypertension patients. The retinal vessels were more likely to be damaged in H-type hypertension than isolated hypertension. However, whether HCY damages retinal vessels through the superposition effect of hypertension or directly damages retinal vessels remains controversial [23, 28]. In short, our research is of great significance for the detection and intervention of plasm HCY in hypertension.

Our study has several potential limitations. There may be manual errors in measuring the diameter of blood vessels even though we tried to avoid that by taking the average value of repeated measurement. It would be better if we could use a newer and more accurate method to analyze the retinal vessel abnormalities. Secondly, when we analyzed, the small sample limited the statistical power of the model. In the future, we need to increase the sample size and conduct cohort studies to better explain these associations and meet the needs of clinical applications.

## Conclusions

We demonstrated that H-type hypertension patients had worse retinopathy compared with hypertension patients, and HCY aggravated lesions of retinal vessels. Controlling H-type hypertension may reduce the risk of small vascular damage.

## Data Availability

The data that support the findings of this study are available from the corresponding author upon reasonable request.

## Abbreviations

HCY: Homocysteine
HHCY: Hyperhomocysteinemia
SBP: Systolic blood pressure
DBP: Diastolic blood pressure
PD: Papilla disc
SD: Standard deviation
IQR: Interquartile range
LDL: Low-density lipoprotein
HDL: High-density lipoprotein
CRVE: Central retinal vein equivalent
CRAE: Central retinal artery equivalent
AVR: Arteriole-to-venule ratio
AVN: Arteriovenous nicking
OR: Odds ratio
CI: Confidence interval
